# Regulation of follicular activation signaling pathways by in vitro inhibition of YAP/TAZ activity in mouse ovaries

**DOI:** 10.1038/s41598-023-41954-0

**Published:** 2023-09-15

**Authors:** Melody Devos, Joana Dias Nunes, Nathalie Donfack Jiatsa, Isabelle Demeestere

**Affiliations:** 1 Research Laboratory on Human Reproduction, Université Libre de Bruxelles (ULB), Campus Erasme CP636, Route de Lennik 808, 1070 Brussels, Belgium; 2 Fertility Clinic, HUB-Erasme Hospital, Université Libre de Bruxelles (ULB), Route de Lennik 808, 1070 Brussels, Belgium

**Keywords:** Ovary, Cell signalling

## Abstract

The Hippo pathway plays a crucial role in the regulation of follicular activation, which constitutes the first step of the folliculogenesis process. Disruption of this pathway occurs in several non-physiological contexts, after fragmentation for ovarian tissue cryopreservation procedures or chemotherapy exposure, leading to massive follicular growth and depletion. This study aimed to investigate the effect of controlling the Hippo pathway using verteporfin (VERT) during in vitro ovarian culture and to evaluate its potential preventive effects on chemotherapy-induced follicle activation using a mouse model. After exposure of cut ovaries to different concentrations of VERT for 3 h, a dose-dependent effect of VERT was observed that reached significant inhibition of YAP activity at 3 µmol/L. To assess the potential effect of controlling chemotherapy-induced Hippo pathway disruption, whole mouse ovaries were exposed to VERT alone or as a co-treatment with 4-hydroperoxycylophosphamide (4HC). VERT co-treatment prevented chemotherapy-induced YAP activation but had a limited impact on downstream effector gene, *Ccn2*. Surprisingly, VERT co-treatment also prevented mTOR and survival signaling pathway alterations following chemotherapy exposure. These results suggest an interaction between the two main signaling pathways regulating follicle activation and a protective effect of VERT on 4HC-induced DNA damage.

## Introduction

The primordial follicle (PF), that constitutes the ovarian reserve, is activated during the first step of follicular growth by transformation of granulosa cells (GCs) from flattened to cuboidal forms to reach the primary follicle stage^1^. This process is irreversible, leading to follicular atresia during growth or ovulation^2^. Adequate regulation of this process is crucial to maintenance of the follicular pool during reproductive life, but it can be disturbed by several non-physiological processes such as in vitro culture or chemotherapy exposure^3^. Follicle activation is regulated by two main intrafollicular signaling pathways, the phosphatidylinositol 3-kinase/protein kinase B/mammalian target of rapamycin (PI3K/AKT/MTOR) pathway and the Hippo pathway^3^. Studies have shown that the PI3K/AKT/MTOR signaling pathway plays a critical role in the control of activation and survival of primordial follicles^4^. Following KIT LIGAND (KL)-cKIT interactions, PI3K phosphorylates phosphatidylinositol-4,5-bisphosphate (PIP2), increasing membrane levels of phosphatidylinositol-3,4,5-trisphosphate (PIP3), and subsequently, inducing the activation of AKT^5^. These events are reversed by the dephosphorylation of PIP3 by the phosphatase and tensin homolog (PTEN) protein^6^. AKT promotes cell survival, and stimulates protein synthesis and cell growth, by activating the MTOR pathway through the inhibition of the heterotrimeric tuberous sclerosis complex 1/2 (TSC1/2)^7,8^. Activated MTOR Complex 1 (MTORC1) leads to the activation of downstream regulators, including 40S ribosomal protein S6 (RPS6) and eukaryotic translation initialization factor 4E (EIF4E), which directly affect protein synthesis and cell growth^9,10^.

The Hippo pathway is a highly conserved signaling pathway that controls organ development through regulation of cell proliferation and apoptosis^11^. This inhibitory pathway is a kinase-regulated network that includes the mammalian Ste20-like kinase 1/2 (MST1/2) and the large tumor suppressor homolog 1/2 (LATS1/2), the downstream effectors yes-associated protein (YAP) and transcriptional coactivator with PDZ-binding motif (TAZ)^12^. Hippo pathway activation results in the phosphorylation of the YAP/TAZ complex, which leads to its cytoplasmic retention and degradation. Consequently, YAP/TAZ nuclear activity is inhibited, and genes involved in cell proliferation are downregulated^13^. Conversely, disruption of the Hippo pathway leads to the accumulation of unphosphorylated YAP/TAZ in the nucleus which, in turn, stimulates the expression of downstream genes such as connective tissue growth factors (*Ctgf* or *Ccn*) and baculoviral inhibitors of apoptosis containing repeats (*Birc*)^11^. Moreover, ovary fragmentation leads to the polymerization of actin, resulting in the nuclear translocation of YAP followed by the production of CCN growth factors and anti-apoptotic BIRC, leading to stimulation of follicle growth^14^.

Previous work from our laboratory highlighted the upregulation of Hippo pathway effectors *BIRC1* and *CCN*2 during ovarian tissue processing for cryopreservation^15^. In human and mouse models, we also reported an increase in follicle activation in vitro through the upregulation of both the PI3K/AKT/MTOR and Hippo pathways^15,16^. Furthermore, a significant decrease of YAP activity was observed after inhibition of mTORC1 in mouse ovaries exposed to chemotherapy, suggesting a possible interaction between the two pathways^16^.

The accelerated follicular activation observed under non-physiological conditions may compromise ovarian reserve or oocyte quality^15,17,18^. Therefore, regulation of the recruitment of quiescent follicles is crucial to preserving ovarian reserve but may further affect oocyte quality and follicular development. Moreover, inhibition of the PI3K/AKT/MTOR pathway has a limited impact on primordial follicle activation^15,19^, suggesting a possible interaction between signaling pathways in the ovary that may contribute to the complexity of follicle recruitment and growth in vitro.

Considering the limited effect of MTORC1 inhibition, the use of verteporfin (VERT) to inhibit the activity of YAP/TAZ might be an interesting approach since it has been demonstrated that this inhibition reduces follicle activation and growth in mouse ovaries^14^. VERT has been identified as an inhibitor of the interaction of YAP with transcriptional enhancer factor (TEAD) which, in turn, blocks transcriptional activation of targets downstream of YAP^20^. One study has shown that pretreatment of mice with VERT blocks *Ccn2* expression induced by ovarian fragmentation^14^. Although not quantified, another study has reported that follicular growth was inhibited after incubating mouse ovaries for 7 days with VERT, suggesting that pharmacological inactivation of YAP in the mouse ovary disrupts follicle development^12^. However, this study reported a high percentage of apoptosis with VERT, highlighting the potential toxicity of this drug.

The aim of this study was to assess the efficacy and toxicity of in vitro exposure to VERT during the early phase of folliculogenesis using a mouse model. We also assessed the potential protective effect of this inhibitor on the chemotherapy-induced impairment of signaling pathways governing activation of ovarian follicles. Finally, we evaluated the possible interaction between the Hippo and PI3K/AKT/MTOR signaling pathways during in vitro follicle activation.

## Results

### Verteporfin prevents fragmentation-induced disruption of the Hippo pathway

**Figure 1:**
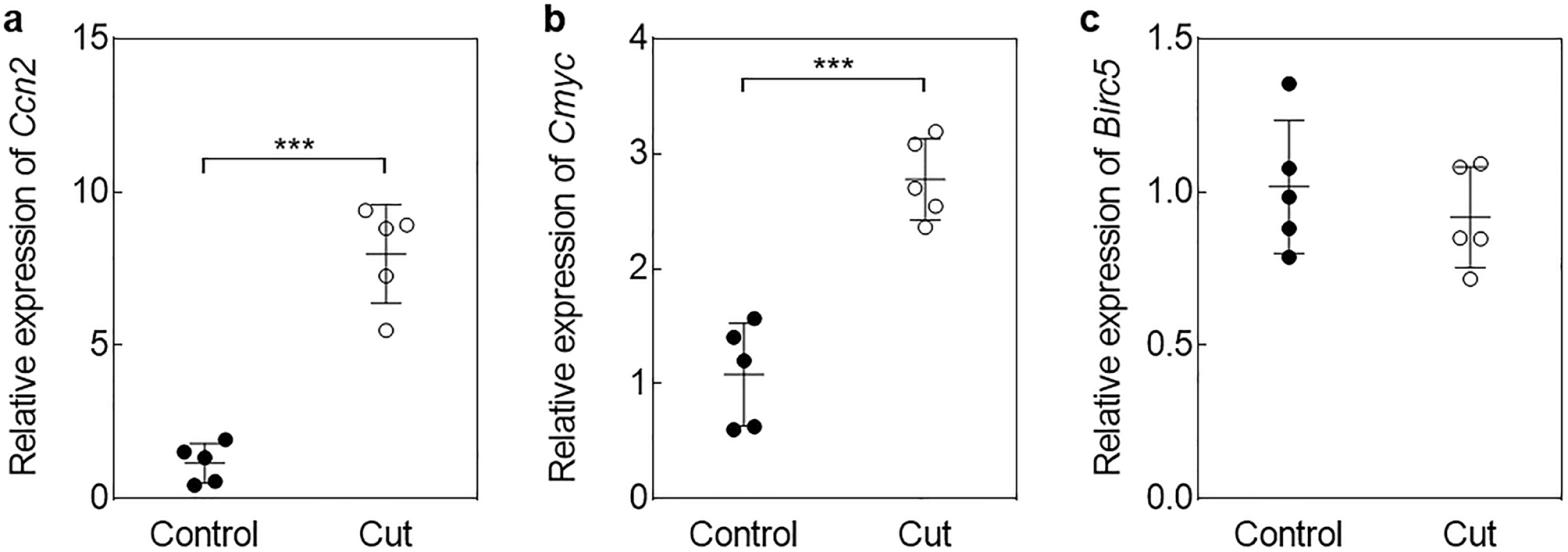
Impact of sectioning on the Hippo signaling pathway after 3 h of culture. Relative expression of (**a**) *Ccn2*, (**b**) *Cmyc*, and (**c**) *Birc5* in whole and cut ovaries. Cycle threshold (Ct) were normalized on *Actin* and *Rpl19* levels and fold-change was obtained based on the Ct mean of intact ovaries group (Control). Data presented are mean ± SD. (N = 5) (***P < 0.001).

The impacts of in vitro culture on follicle activation were previously demonstrated using newborn mouse ovaries and human cortical tissues^15,16^. Other studies have also shown that murine ovarian fragmentation leads to disruption of the Hippo pathway, resulting in upregulation of its downstream effectors, such as *Ccn2,3,5,6* and *Birc1,7* from 2 h post-sectioning^14^. In this study, Hippo pathway disruption was confirmed after 3 h of in vitro culture with a higher expression of *Ccn2* (*P* < 0.001) and *Cmyc* (*P* < 0.001) in cut ovaries compared to whole controls, while *Birc5* expression was not affected following sectioning (*P* = 0.437) (Fig. 1a-c). Expression of *Kl* was significantly higher in cut ovaries compared to controls (*P* = 0.014), while *Tsc1* (*P* = 0.946) and *Mtor* (*P* = 0.095) expression remained stable among groups (Supplementary Figure S1a-c). Similarly, gene expression levels of *Bcl-2–associated X* (*Bax*) (*P* = 0.096) and *B-cell lymphoma 2* (*Bcl2*) (*P* = 0.095) were similar among groups (Supplementary Figure S2a,b).

**Figure 2:**
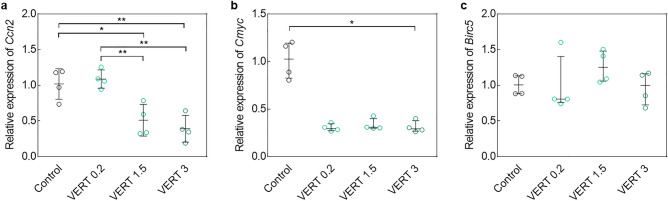
Effect of verteporfin (VERT) treatment on the Hippo signaling pathway after 3 h of culture following sectioning. Relative expression of (**a**) *Ccn2*, (**b**) *Cmyc*, and (**c**) *Birc5* in cut ovaries exposed to 0.2, 1.5, and 3 µmol/L VERT. After normalization on *Actin* and *Rpl19* levels, fold-change was obtained based on the Ct mean of the untreated ovaries group (Control). Data are mean ± SD (*Ccn2*) and median ± interquartile range (IQR) (*Cmyc* and *Birc5*). (N = 4) (*P < 0.05; **P < 0.01).

To assess the ability of VERT to prevent the impact of sectioning, half-cut ovaries were cultured for 3 h and with different concentrations of VERT (0.2, 1.5, and 3 µmol/L). A significant decrease in *Ccn2* expression was observed in ovaries treated with 1.5 and 3 µmol/L VERT compared to controls (*P* = 0.013; *P* = 0.003) and to 0.2 µmol/L VERT (*P* = 0.005; *P* = 0.001) conditions (Fig. 2a). Although *Birc5* levels did not vary among groups, *Cmyc* expression was lower in ovaries cultured with VERT compared to controls reaching significance at 3 µmol/L (*P* = 0.045) (Fig. 2b,c). *Kl* expression was significantly lower in 1.5 µmol/L treated ovaries compared to controls (*P* = 0.036) but the difference did not reach significance in the other concentrations. *Tsc1* and *Mtor* levels were not significantly different among cultured groups (Supplementary Figure S3a-c). Expression levels of *Bax* and *Bcl2* were stable following VERT treatment, irrespective of the concentration (Supplementary Figure S4a,b).

### Verteporfin prevents chemotherapy-induced follicular damage in vitro

Our previous study showed that 4HC exposure impacts both PI3K and Hippo signaling pathways in neonatal mouse ovaries, leading to follicle activation^16^. To assess the potential effect of VERT during 4HC exposure to control signaling pathway alterations, the dose of 3 µmol/L of VERT was used. To preserve 4HC-induced follicle activation and limit its gonadotoxicity, a concentration of 10 µmol/L of chemotherapeutic agent was chosen for this study based on previous experiments^16,21^. The effect of VERT on chemotherapy-induced signaling pathways was investigated at 24 and 48 h of culture with 24-h exposure to treatments.

**Figure 3:**
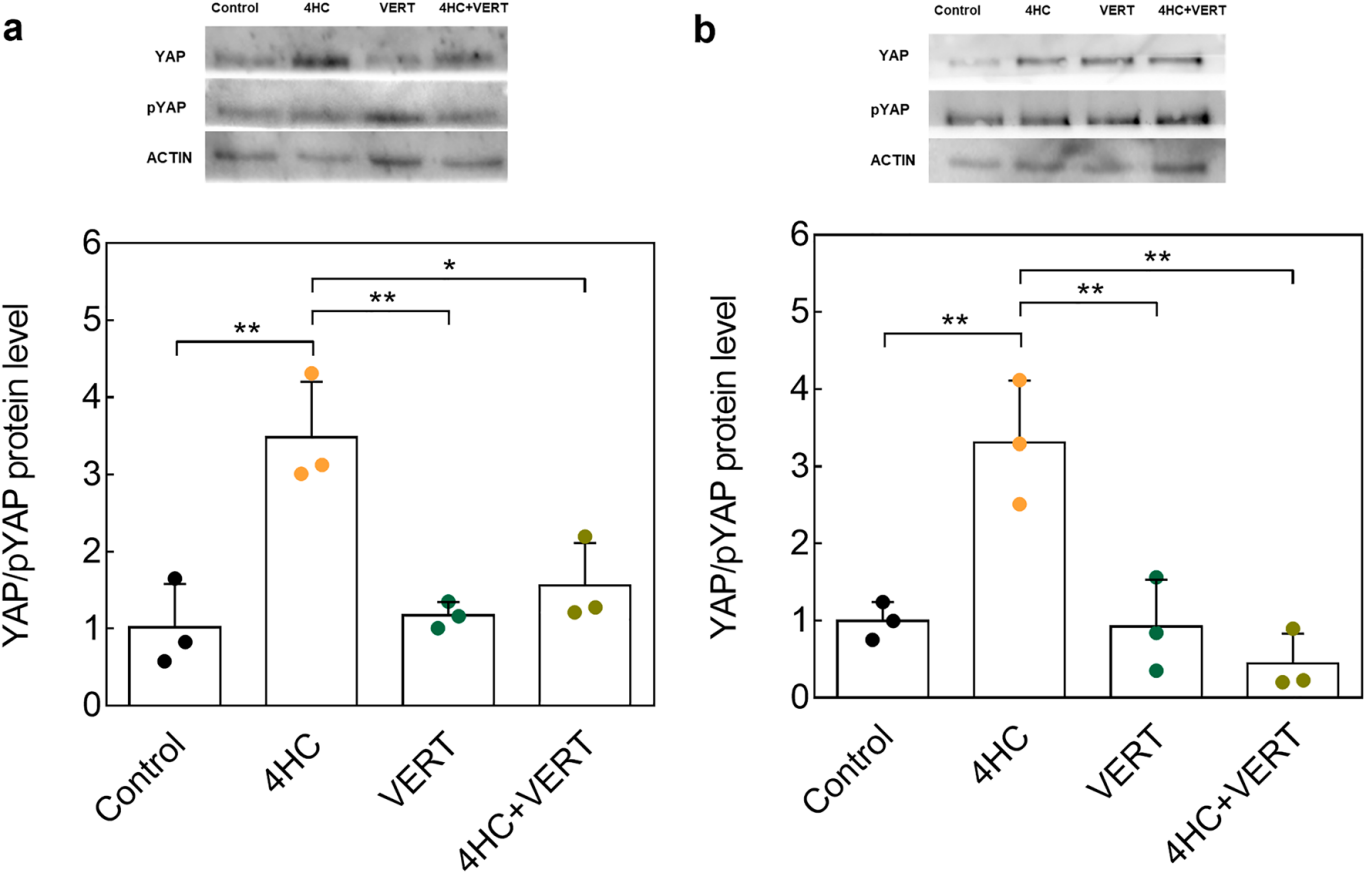
Protein analyses of the Hippo pathway following 4HC and VERT treatment of whole ovaries. (**a**) Western blot images and protein ratio levels of YAP and pYAP after 24 h of culture among the different conditions. (**b**) Images of western blot and quantification of the YAP/pYAP ratio after 48 h of culture. Quantification was performed after normalization on ACTIN levels and on the control condition. Values represented are mean ± SD. (N = 3). (*P < 0.05; **P < 0.01). VERT, Verteporfin; 4HC, 4-hydroperoxycyclophosphamide.

**Figure 4:**
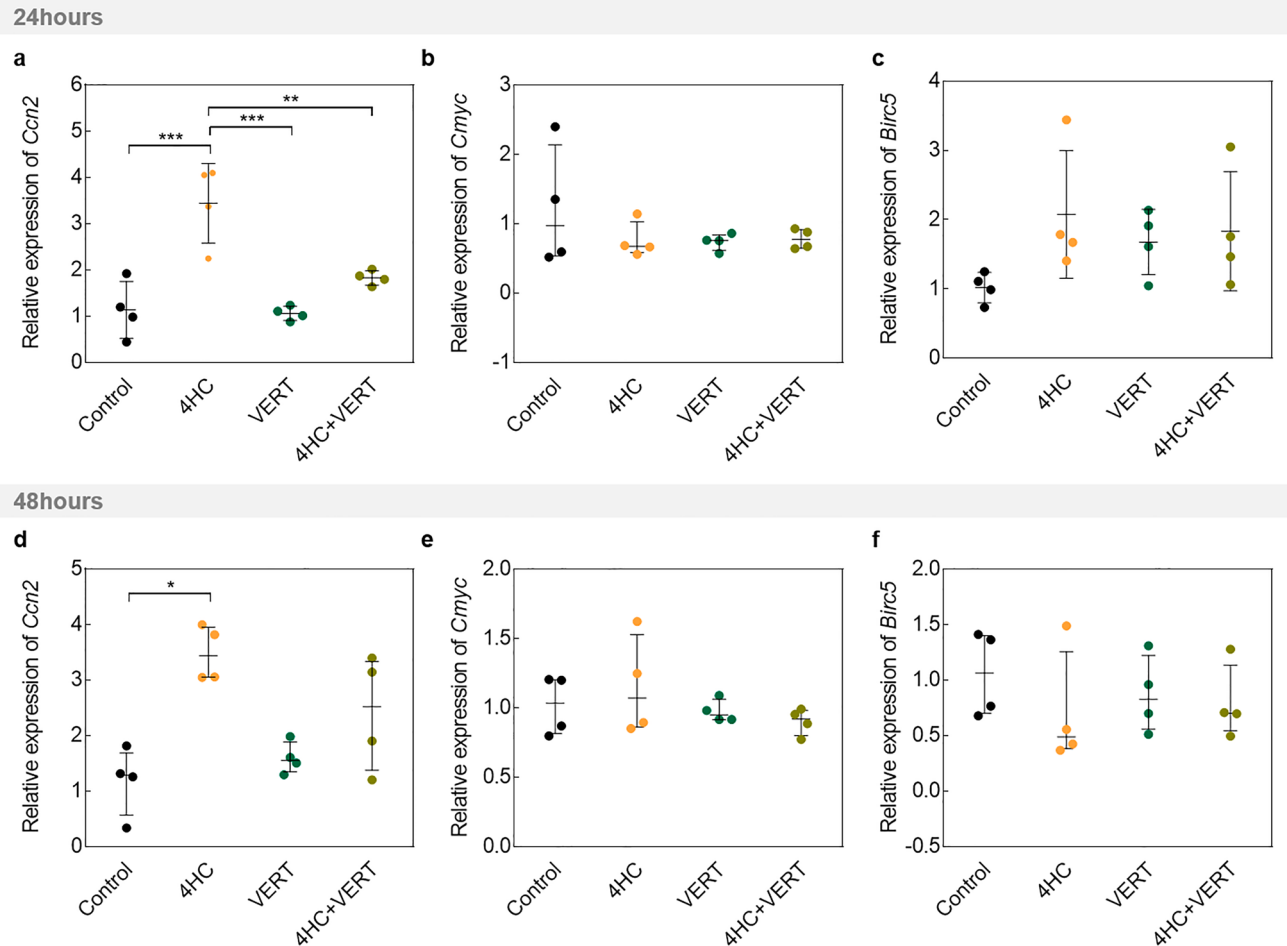
Analyses of genes regulated by the Hippo pathway after 24 and 48 h of culture in intact ovaries exposed or not exposed to 10 µmol/L 4HC and/or 3 µmol/L VERT. Relative expression of (**a**) *Ccn2*, (**b**) *Cmyc*, and (**c**) *Birc5* after 24 h of culture. Data are mean ± SD (*Ccn2* and *Birc5*) and median ± interquartile range (IQR) (*Cmyc*). Gene expression of (**d**) *Ccn2*, (**e**) *Cmyc*, and (**f**) *Birc5* after 48 h of culture among the different conditions. Data are median ± IQR. Normalization was performed on *Actin* and *Rpl19* levels and on the mean of the untreated group (Control). (N = 4) (*P < 0.05; **P < 0.01; ***P < 0.001). VERT, Verteporfin; 4HC, 4-hydroperoxycyclophosphamide.

We first assessed the impact of co-treatment on the Hippo signaling pathway by protein and gene analyses (Figs. 3, 4). A significantly higher protein ratio of YAP/pYAP was observed in 4HC-treated ovaries compared to controls after 24 (*P* = 0.002) and 48 (*P* = 0.004) hours of culture (Fig. 3a,b). At both culture timepoints, ovaries cultured with the co-treatment had a significantly lower YAP/pYAP ratio compared to ovaries exposed to 4HC alone (24 h, *P* = 0.01; 48 h, *P* = 0.001) (Fig. 3a,b). Exposure to 4HC increased *Ccn2* gene expression after 24 h of culture compared to controls (*P* < 0.001) and co-treatment with VERT significantly decreased its expression compared to 4HC alone (*P* = 0.006) (Fig. 4a). Similarly, after 48 h of culture, 4HC exposure resulted in a higher level of *Ccn2* expression compared to controls (*P* = 0.036), but no preventive effect of the co-treatment was observed (Fig. 4d). Although some trends were observed between conditions, no significant effects on other Hippo pathway gene effectors were observed after exposure to chemotherapy alone or in co-treatment at both culture timepoints (Fig. 4b,c,e,f).

**Figure 5:**
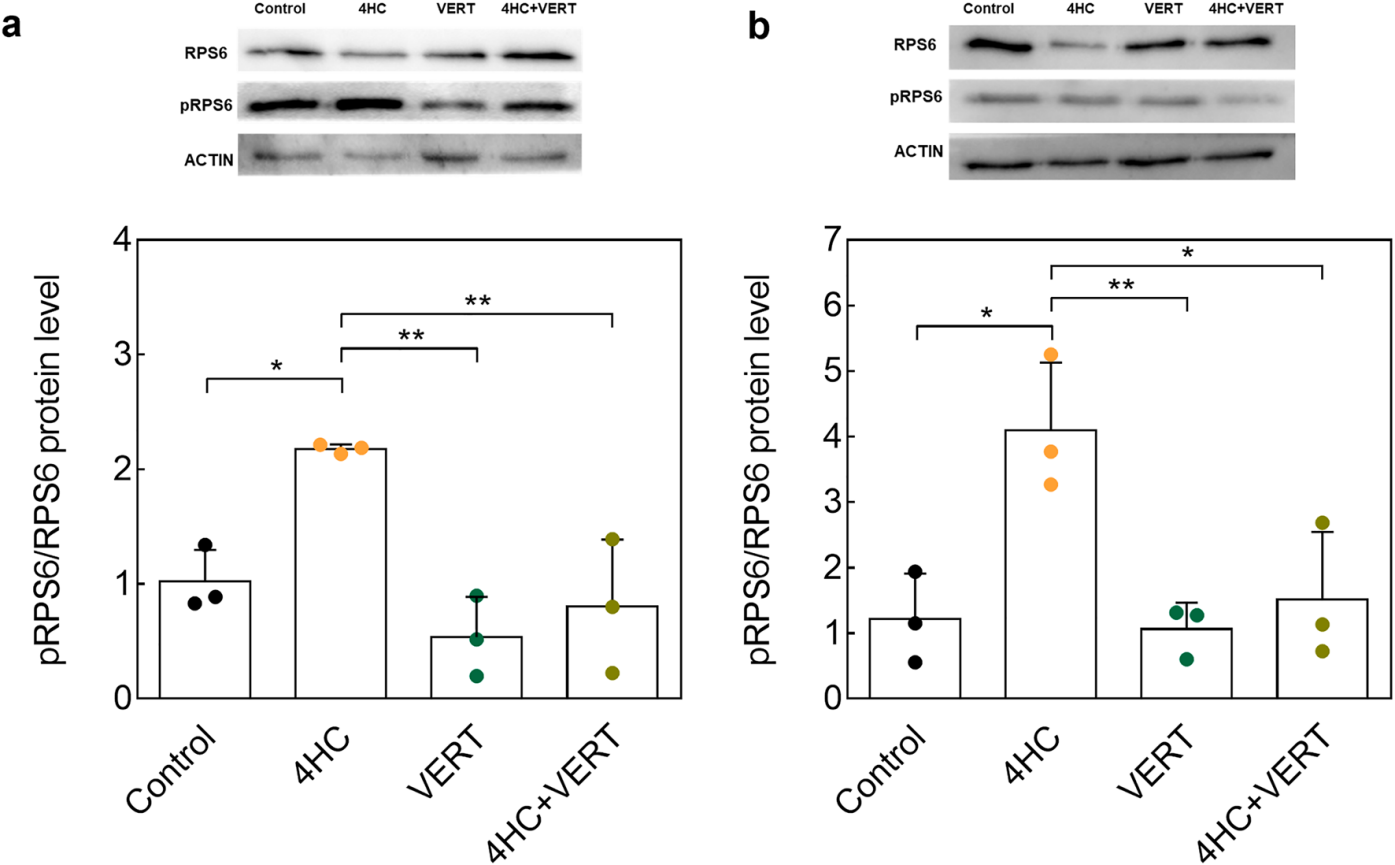
Effect of the co-administration of VERT with 4HC on PI3K protein levels after 24 and 48 h of culture. Western blot images and protein quantification of pRPS6/RPS6 ratio after (**a**) 24 h of culture and (**b**) 48 h of culture. Data are presented with mean ± SD. Normalization was performed on ACTIN level and on the untreated group (Control). (N = 3) (*P < 0.05; **P < 0.01). VERT, Verteporfin; 4HC, 4-hydroperoxycyclophosphamide.

We further assessed the PI3K signaling pathway through gene analyses of *Kl*, *Tsc1,* and *Mtor* and observed no variations among the different conditions at both analysis times (Supplementary Figure S5a-f). The impact of co-treatment on PI3K proteins was then evaluated by western blotting for AKT and RPS6, as well as their phosphorylated forms (Fig. 5). No significant differences in pAKT/AKT ratio were observed among the different conditions at both culture times (*data not shown*). Ovaries exposed to 4HC alone had significantly higher pRPS6/RPS6 levels compared to controls at 24 (*P* = 0.02) and 48 (*P* = 0.012) hours of culture. When ovaries were exposed to VERT co-treatment with 4HC, we observed a significant decrease in this ratio compared to the 4HC alone condition (24 h, *P* = 0.008; 48 h, *P* = 0.022) (Fig. 5a,b).

**Figure 6:**
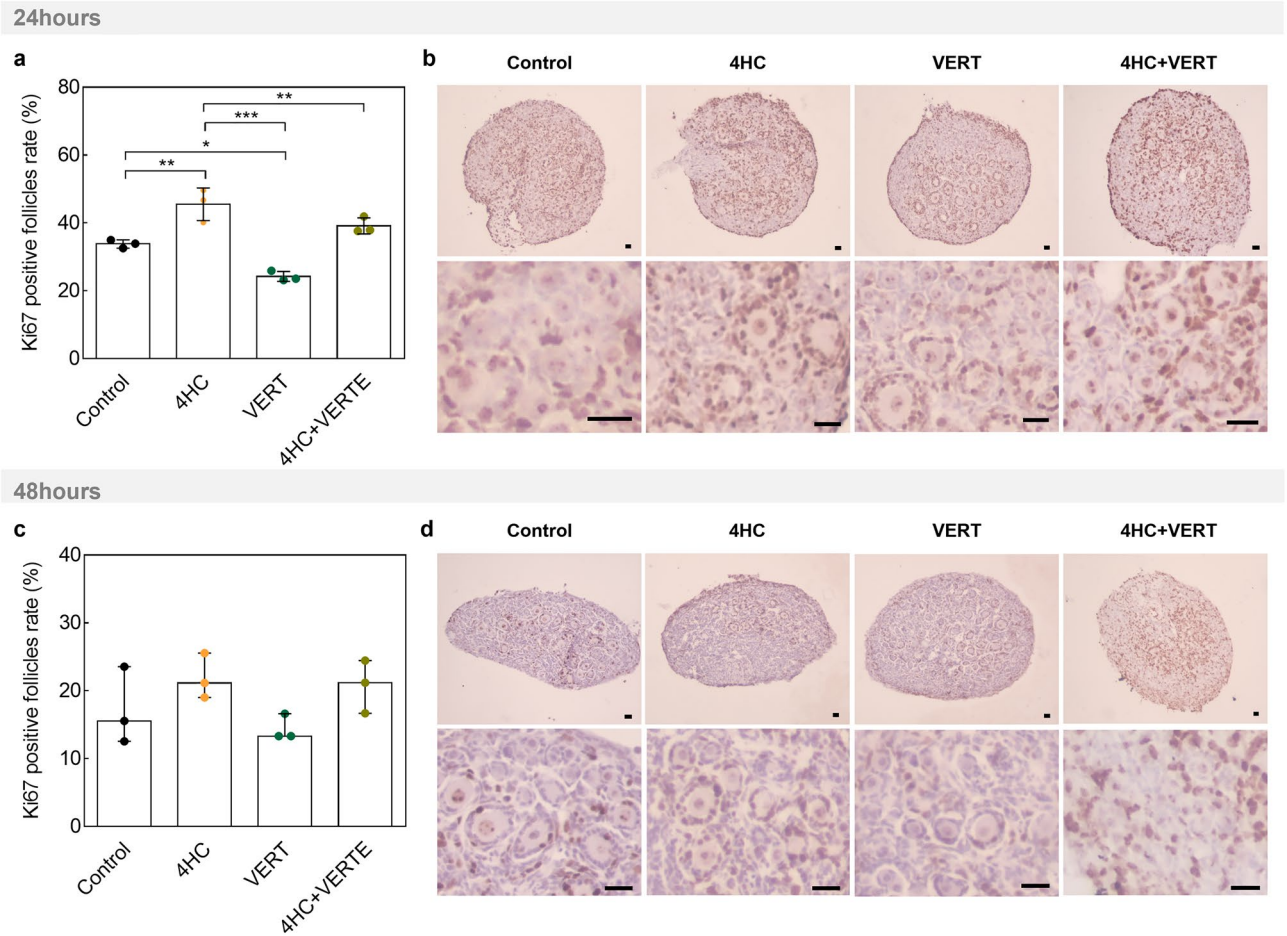
KI67 staining after 24 and 48 h of culture on sections from ovaries exposed to 4HC and/or VERT. (**a**) Quantification of KI67-positive follicles among the conditions after 24 h of culture. Data are mean ± SD. (**b)** Images of KI67 staining after 24 h of culture. (**c**) Quantification of KI67-positive follicles after 48 h of culture. Data are median ± interquartile range (IQR). (**d**) KI67 staining pictures among the different groups after 48 h of culture. Scale bar = 20 µm. (N = 3) (*P < 0.05; **P < 0.01; ***P < 0.001). VERT, Verteporfin; 4HC, 4-hydroperoxycyclophosphamide.

To assess the impact of the different treatments on follicle activation, staining of KI67, a cell proliferation marker, was performed and healthy positive follicles were counted at both time of culture (Fig. 6a-d). After 24 h of culture, ovaries exposed to 4HC contained significantly higher positively-stained follicles than control ones (*P* = 0.005) (Fig. 6a,b). VERT exposure showed significant lower levels of proliferation rate compared to control (*P* = 0.014) and 4HC (*P* < 0.001) conditions. VERT co-treatment appeared to prevent the 4HC-induced proliferation (*P* = 0.001) (Fig. 6a,b). After 48 h of culture, although similar trends were observed, KI67 staining appeared to be lower than at 24 h and non-significantly different among the conditions (Fig. 6c,d).

**Figure 7:**
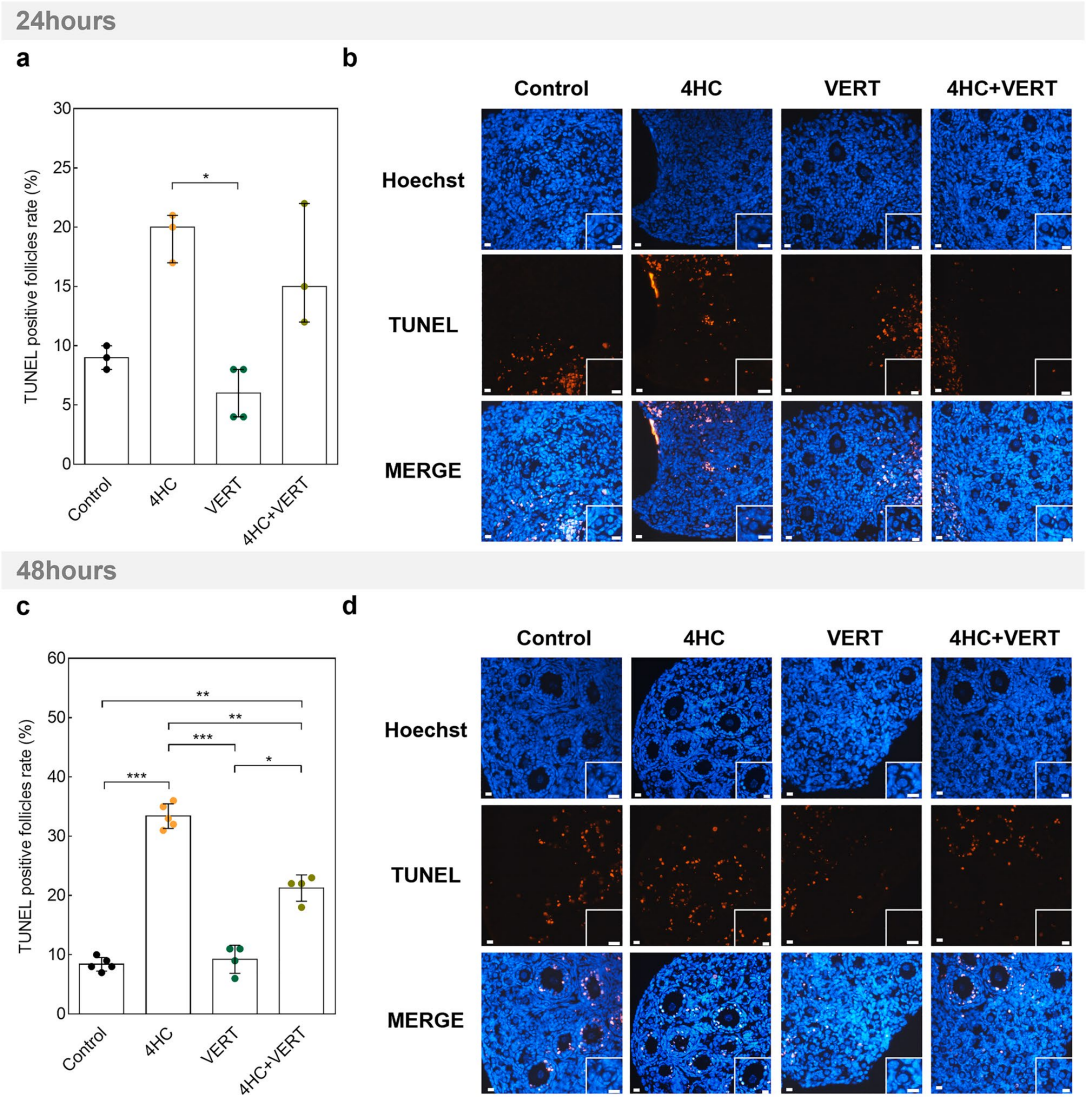
TUNEL staining among ovaries treated with 4HC and/or VERT after 24 and 48 h of culture. (**a**) Quantification of TUNEL-positive follicles among the groups after 24 h of culture. Data are median ± interquartile range (IQR). (**b)** Images of TUNEL staining on different conditions after 24 h of culture. (**c**) Quantification of TUNEL-positive follicles after 48 h of culture. Data are mean ± SD. (**d**) TUNEL staining pictures among the different conditions after 48 h of culture. Scale bar = 20 µm. (N = 3–5) (*P < 0.05; **P < 0.01; ***P < 0.001). VERT, Verteporfin; 4HC, 4-hydroperoxycyclophosphamide.

Finally, VERT toxicity was assessed through apoptosis gene analyses and DNA damage staining (Fig. 6, Supplementary Figure S6). The expression levels of *Bax* and *Bcl2* were stable among the conditions, except that a higher level of *Bax* was observed after 48 h of culture in 4HC-exposed ovaries compared to controls (*P* = 0.036) (Supplementary Figure S6a-d). DNA damage staining analysis showed no deleterious impacts of VERT exposure in comparison with control conditions at both culture timepoints (Fig. 7a–d). While 4HC induced apoptosis at 48 h of culture (*P* < 0.001), VERT significantly prevented this effect (*P* = 0.002) (Fig. 7c,d). However, the percentage of positive follicles remained higher in the co-treatment conditions compared to control (*P* = 0.002) and VERT (*P* = 0.017) conditions (Fig. 7c,d).

## Discussion

One of the main concerns regarding ovarian tissue processing remains the uncontrolled massive follicle activation associated with the process. Previous studies in mouse and human models have reported an increase in growing follicles following processing and in vitro culture^15,16^. Using a newborn mouse model, our laboratory reported that organ removal is sufficient to disrupt the Hippo signaling pathway^16^. Fragmentation of mouse ovaries has been reported to disrupt the Hippo pathway through modulation of actin polymerization and leads to increased expression of *Ccn* and *Birc* genes from 2 h post-sectioning^14^. Moreover, we have also reported that sectioning impairs both PI3K and Hippo signaling after 4 h of culture^16^. Non-physiological activation can compromise vital oocyte–somatic cell contacts and, consequently, oocyte quality^17^. A possible option to reduce this effect is the use of inhibitors to control signaling pathways involved in follicle activation. Therefore, we investigated the efficacy of pharmacological control of the Hippo signaling pathway using VERT, an inhibitor of YAP/TAZ activity^22^. Cell culture studies have reported significant repression of *Ccn2* gene expression following exposure to 0.25 µmol/L VERT^23^. Therefore, we first exposed cut ovaries to 0.2, 1.5, and 3 µmol/L of VERT for 3 h. Following ovarian sectioning, we confirmed a disruption of Hippo signaling and reported a significant downregulation of *Ccn2* and *Cmyc* gene expression in cut ovaries exposed to the highest tested concentration of VERT. Consistent with our previous study, *Birc5* gene expression did not vary following fragmentation or VERT treatment, suggesting that this gene might be controlled by other transcription factors or signaling pathways in the ovary^16^. Notably, YAP/TAZ are known to also interact with mothers against decapentaplegic homologs (SMADs) and p73 factors^24–26^. Moreover, redundancies have been reported between Hippo activity and other signaling pathways such as the nuclear factor kappa-light-chain-enhancer of activated B cells (NFKB) pathway and the WNT-BETA-CATENIN pathway^25,27–29^. Interestingly, we observed that sectioning induced a significantly higher level of *Kl* that appeared to be prevented in VERT-treated ovaries, suggesting a potential link between the Hippo and PI3K pathways through gene modulation of the major trigger of follicle activation^30^.

We also investigated the effect of chemotherapy on the two signaling pathways and the possible benefit of co-treatment with VERT. Hippo pathway analyses revealed an increase in YAP levels following 4HC exposure, as previously reported, and a significant prevention of this increase when ovaries when co-treated with VERT. Consistent with the protein activity, *Ccn2* expression was higher after chemotherapy exposure and downregulated when VERT was used in co-treatment with 4HC compared to 4HC alone. Interestingly, this preventive effect appeared to be present after 24 h of culture but was moderated at 48 h of culture, suggesting that VERT is poorly effective when culture-induced follicular activation has already occurred. We also reported that exposure to VERT alone did not impact the Hippo pathway when the ovaries were not fragmented. These results suggest that VERT activity is effective when Hippo is disrupted, due to sectioning or to 4HC exposure and to the culture. Studies performed in mouse models have reported an increased activation of PI3K signaling after cyclophosphamide injection or 4HC exposure^16,31,32^. Our previous study reported an induction of pAKT and pRPS6 after 4 h of exposure to 4HC at 3 and 20 µmol/L, that decreased with time, with pRPS6 levels still slightly increased after 48 h of culture^16^. Consistent with our previous report, we observed an increase in RPS6 activation in ovaries exposed to 10 µmol/L 4HC compared to controls at both culture timepoints.

Previous studies have suggested regulation of Hippo signaling by PI3K pathway components^16,33^. Notably, we reported in an in vitro mouse model that co-treatment with a mTORC1 inhibitor, everolimus, prevents Hippo pathway disruption following chemotherapy exposure^16^. Interestingly here, VERT co-treatment resulted in a significant decrease in pRPS6/RPS6 levels in comparison with the 4HC condition. This result suggests a second interaction mediated by Hippo effectors on PI3K signaling, indicating a potential bi-directional crosstalk between these pathways.

The PI3K and Hippo signaling pathways are known to control cellular fate and survival^7,26,34^. Moreover, the gonadotoxicity of 4HC on mouse ovarian tissue has been well demonstrated^16,21,31^. Therefore, we also assessed the impact of VERT treatment on follicle growth and survival in our short-culture system. We first observed an acute induction of proliferation among healthy follicles following 4HC exposure, prevented by co-treatment with VERT. We then confirmed that DNA damage levels increased significantly after exposure to 4HC but only observed a slight protective effect of the co-treatment after 48 h of culture. This prevention of DNA damage appeared to be limited as the level of DNA breaks remained higher in the 4HC-treated condition than in the control condition. Altogether, these two results suggest an acute and deleterious follicle entrance into growth phase following culture with 4HC, that can partially be prevented with VERT.

Overall, this in vitro culture study confirmed that the PI3K and Hippo pathways are dysregulated by ovarian tissue sectioning and chemotherapy exposure. Our results underscore the fact that VERT treatment can control Hippo pathway disruption following sectioning, and can partially prevent the impairment of the MTOR and Hippo pathways following chemotherapy exposure at the concentrations we used. Our data further support an interaction between the two pathways to control follicle activation in non-physiological conditions. Despite the fact that a limited protective effect of VERT against chemotherapy damage was observed, the use of this inhibitor could be a promising approach in association with other inhibitors to effectively control both signaling pathways. The limited time of exposure and culture in our study should be considered and results should be interpreted with caution as the effect seems to be transitory.

## Materials and methods

### Experimental model

Adult CBA/Ca male and C57Bl/6 female mice from Janvier Laboratory were housed in a controlled environment (Specific-Pathogen-Free [SPF], 12-h light/dark cycle, standard temperature, food and water provided ad libitum). Experiments were performed using F1 mice (CBA/Ca x C57Bl/6) at day 3 or 4 of age. All experimental procedures were carried out with prior agreement of the Animal Welfare Ethics Committee of the Medicine Faculty at the Université libre de Bruxelles (ULB, Belgium).

### In vitro culture

Female post-natal day 3 or 4 (PND3/4) mice were sacrificed by decapitation and ovaries were collected and processed as previously described^16^. To assess VERT efficacy on YAP function inhibition, whole and half-cut ovaries were exposed to 0.2, 1.5, and 3 µmol/L of VERT (Sigma) and cultured in vitro for 3 h. The effect of VERT on chemotherapy-induced follicle activation was evaluated after 24 or 48 h of culture. Intact ovaries were exposed for 24 h to 3 µmol/L of VERT alone or as a co-treatment with 4-hydroperoxycyclophosphamide (4HC; Sigma), an active metabolite of cyclophosphamide, used at 10 µmol/L. For experiments at 48 h of culture, VERT and/or 4HC were added at 24 h with media replacement. During each experiment, a control condition was obtained by exposing ovaries to 0.1% dimethylsulfoxide (Sigma).

### Histological analysis

Ovaries were fixed in 4% paraformaldehyde at 4 °C for 2 h after the culture. Samples were dehydrated in increasing concentrations of ethanol before paraffin embedding, then serially sectioned. Cell proliferation was analyzed by KI67 staining (BD Bioscience, 556,003), as previously described^16^. At least three sections per ovary from three different experiments were quantified. Morphologically healthy follicles containing at least one stained granulosa cell were considered as positive and reported on the total number of healthy follicles (n ≥ 180 follicles) per ovary. DNA fragmentation was assessed by TdT-mediated dUTP-biotin nick-end labelling (TUNEL) staining, using the In Situ Cell Death Detection Kit (Roche). Sections were deparaffinized, rehydrated, permeabilized with 20 μg/ml proteinase K (Qiagen) in 10 mmol/L Tris pH 7.4 and incubated, according to the manufacturer’s instructions, with TUNEL reagents and counterstained with 1 µg/mL Hoechst. Positive and negative controls were obtained by treating sections with 50 IU/mL DNase I (Invitrogen) and enzyme-free solution, respectively. Follicles were considered positive when containing at least one stained granulosa cell or a stained oocyte. To quantify apoptosis within ovaries, the ratio of positive follicles stained for TUNEL to the total number of follicles (n ≥ 40 follicles) per slide was calculated.

### Western blot

For protein expression and quantification, 8 mouse ovaries were required per condition and protein lysates were obtained by mixing the ovaries with Laemmli buffer solution containing 10% glycerol, 5% beta-mercaptoethanol, 62.5 nmol/L Tris–HCl pH 6.8, 2% sodium dodecyl sulfate (SDS), and protease (Thermo Fisher) and phosphatase (Roche) inhibitors. A 5%-12% SDS–polyacrylamide gel electrophoresis was performed to separate proteins, followed by transfer to nitrocellulose membranes (Bio-Rad). After blocking of non-specific sites, membranes were incubated with 1:500 targeted primary antibodies (ACTIN, pRPS6, pYAP, RPS6, YAP) overnight and with 1:1000 horseradish peroxidase-conjugated secondary antibody^16^. Protein bands were revealed by chemiluminescence using SuperSignal West Femto Maximum Sensitivity Substrate (ThermoFisher) and ChemiDoc XRS + instrument (Bio-Rad). Visualization and quantification of protein bands were performed with Image Lab 3.0 software (Bio-Rad) and ImageJ software, respectively.

### Gene expression analysis

RNA extraction was performed using RNAqueous-Micro Total RNA Isolation Kit (Life Technologies) according to the manufacturer’s instructions. RNA concentration was determined by spectrophotometry using NanoDrop 2000 (Thermo Scientific) and cDNA synthesis was carried out with GoScript Reverse Transcription Mix (Promega). Real-time qPCR was performed in 96-well plates on a 7500 Real-Time PCR System (Applied Biosystems) using Power SYBR Green Master Mix (Applied Biosystems). Primers used were previously validated^16,35^. Target gene transcripts were quantified using the comparative cycle threshold (Ct) method (ΔΔCt) through normalization of the mean Ct with housekeeping genes level (*actin* and 60S ribosomal protein L19–*rpl19*) and control condition. Gene expression levels were evaluated based on the foldchange (2^−ΔΔCt^).

### Statistical analysis

All experiments were conducted at least three times. Depending on normality and homogeneity of variances of our data, parametric and non-parametric tests were performed by Student’s t-test or one-way ANOVA, and Wilcoxon-Mann–Whitney or Kruskal Wallis test, respectively. When required, multiple comparisons were performed with Tukey correction. Variables tested with parametric tests are shown with the mean and the standard deviation (SD). Median and interquartile range (IQR) are represented when non-parametric tests were performed. Statistical analyses were performed using SPSS Statistics 25 (IBM) and graphs were designed with Prism 6 (GraphPad). Statistical significance was considered when *P*-value < 0.05.

### Supplementary Information

Supplementary Information.

## Data Availability

Data used in this study are available from the corresponding author upon reasonable request.
