# Supplementary material for: Regulation of follicular activation signaling pathways by in vitro inhibition of YAP/TAZ activity in mouse ovaries
Source: Sci Rep. 2023 Sep 15;13:15346. doi: 10.1038/s41598-023-41954-0 (PMC10504383; doi:10.1038/s41598-023-41954-0)
Supplement: Supplementary file 1 — Supplementary Information. [file 41598_2023_41954_MOESM1_ESM.pdf]

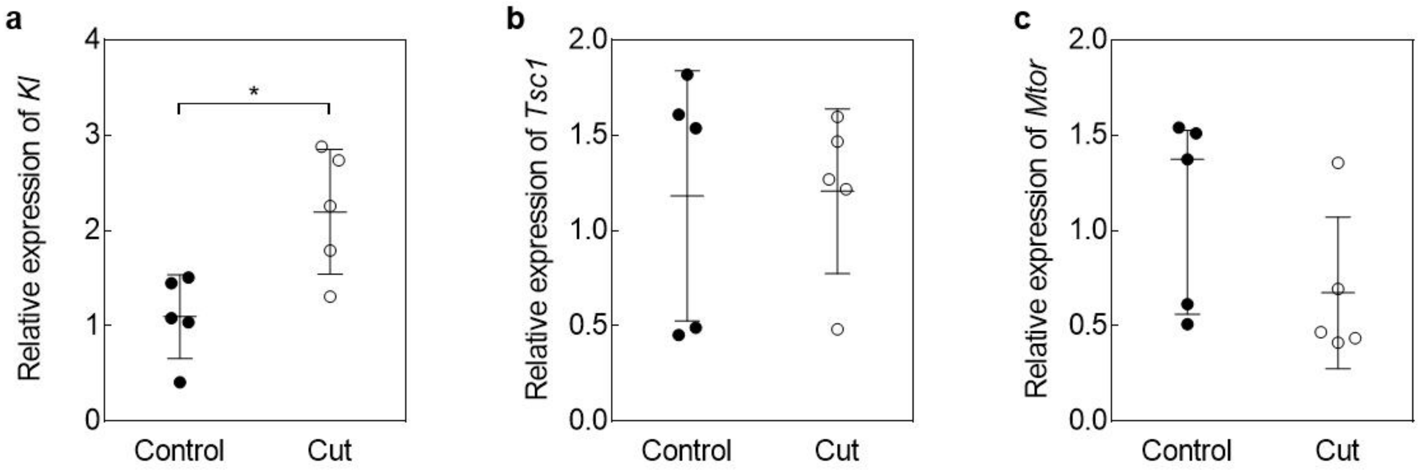

Supplementary Figure S1: Assessment of the impact of sectioning on PI3K signaling pathway genes after 3 hours of culture.

Relative expression of (**a**) *Kl*, (**b**) *Tsc1*, and (**c**) *Mtor* in whole and cut ovaries. Ct were normalized on *Actin* and *Rpl19* levels and fold-change was obtained on the mean of whole ovaries group (Control). Data presented are mean ± SD (*Kl* and *Tsc1*) and median ± interquartile range (IQR) (*Mtor*). (N = 5) (*, $p < 0.05$).

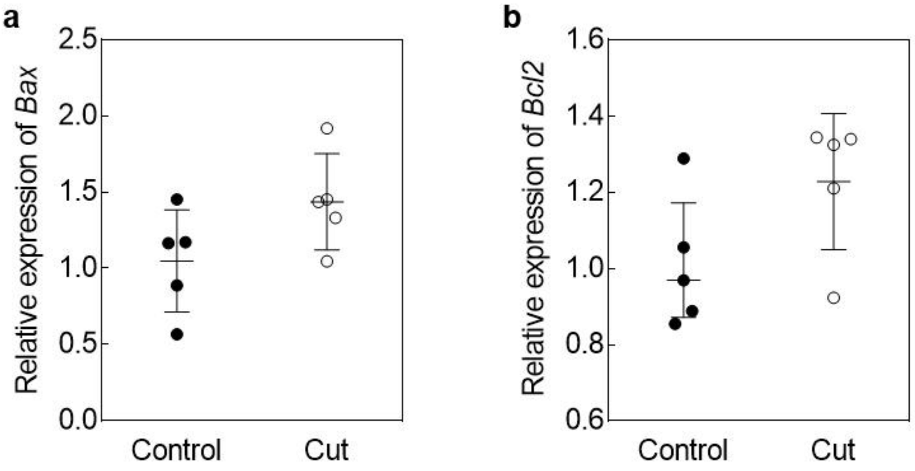

Supplementary Figure S2: Impact of sectioning on survival pathway genes after 3 hours of culture.

Relative expression of (**a**) *Bax* and (**b**) *Bcl2* in intact and half-cut ovaries. Normalization was done on *Actin* and *Rpl19* levels and on the mean of Control group. Data presented are mean ± SD (*Bax*) and median ± interquartile range (IQR) (*Bcl2*). (N = 5)

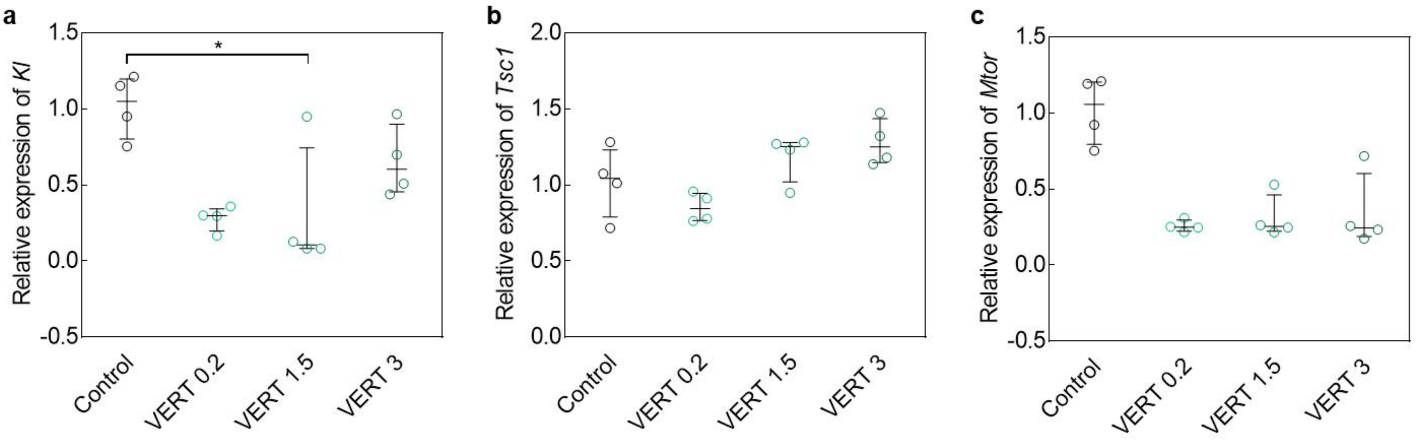

Supplementary Figure S3: Effect of verteporfin (VERT) treatment on PI3K signaling pathway genes after 3 hours of culture following sectioning.

Relative expression of (**a**) *Kl*, (**b**) *Tsc1*, and (**c**) *Mtor* in cut ovaries treated or not with 0.2, 1.5, and 3 µmol/L VERT. After normalization on *Actin* and *Rpl19* levels, fold-change was obtained on the mean of untreated ovaries group (Control). Data are median ± interquartile range (IQR). (N = 4) (*, $p < 0.05$).

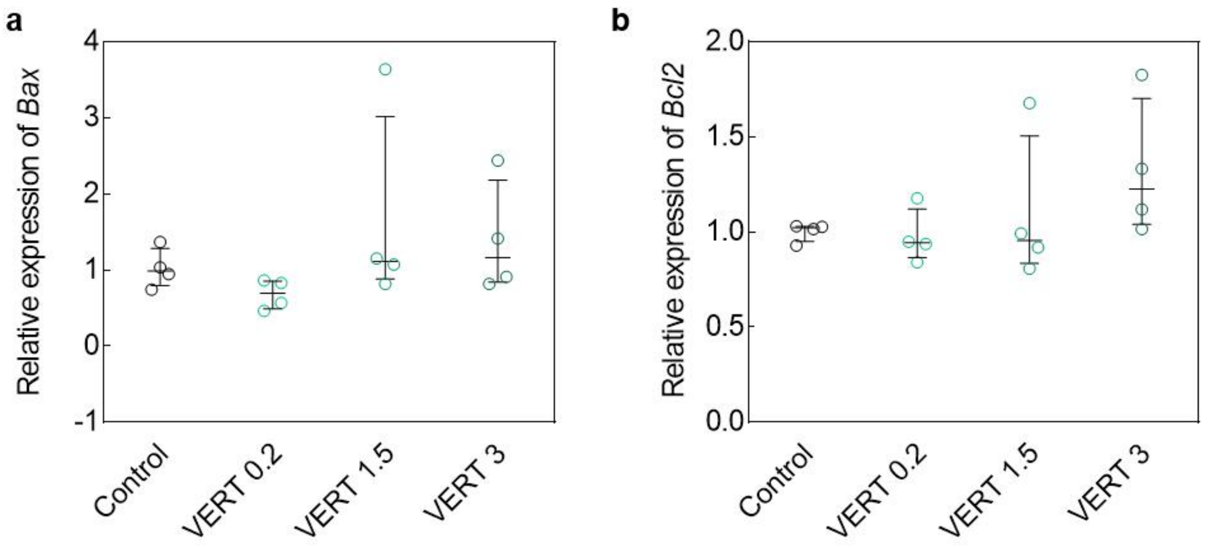

Supplementary Figure S4: Effect of verteporfin (VERT) treatment on survival pathway genes after 3 hours of culture following sectioning.

Relative expression of (**a**) *Bax* and (**b**) *Bcl2* in cut ovaries treated or not with 0.2, 1.5, and 3 µmol/L VERT. Data were normalized on *Actin* and *Rpl19* levels, and on the mean of Control group. Data are median ± interquartile range (IQR). (N = 4).

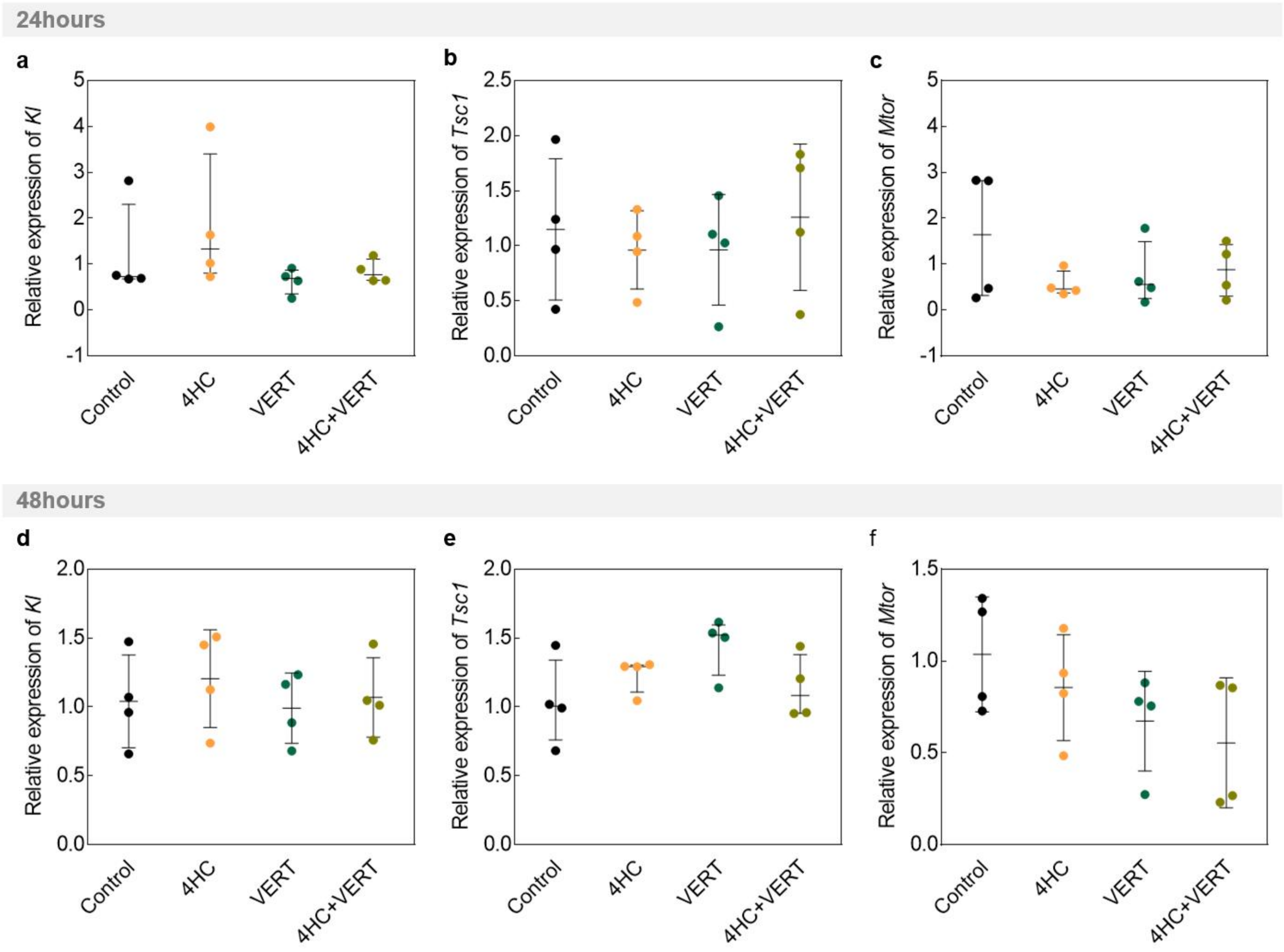

Supplementary Figure S5: PI3K signaling pathway gene analyses after 24 and 48 hours of culture in ovaries exposed to 10 μmol/L 4HC and/or VERT 3μmol/L.

Relative expression of (**a**) *KitLigand (Kl)*, (**b**) *Tsc1*, and (**c**) *Mtor* after 24 hours of culture. Data are mean ± SD (*Tsc1*) and median ± interquartile range (IQR) (*Kl* and *Mtor*). Expression level of (**d**) *Kl*, (**e**) *Tsc1*, and (**f**) *Mtor* after 48 hours of culture. Data presented are mean ± SD (*Kl* and *Mtor*) and median ± IQR (*Tsc1*). After normalization on *Actin* and *Rpl19* levels, fold-change was obtained on the mean of the untreated culture group (Control). (N = 4) VERT, Verteporfin; 4HC, 4-hydroperoxycyclophosphamide.

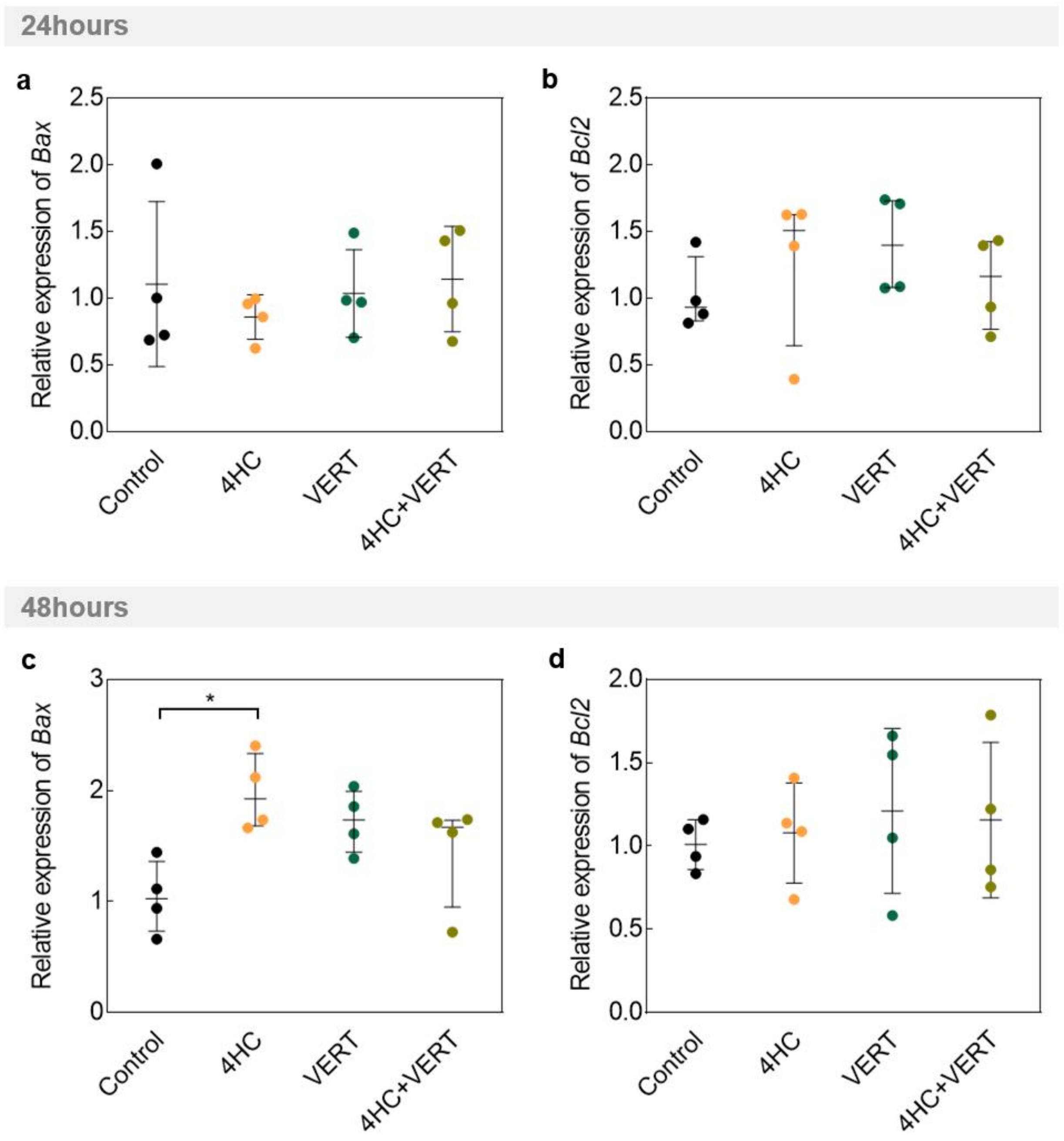

Supplementary Figure S6: Impact of the co-administration of VERT with 4HC on survival pathway genes after 24 and 48 hours of culture.

Relative expression of (**a**) *Bax* and (**b**) *Bcl2* after 24 hours of culture among untreated ovaries (control), ovaries exposed to 10 µmol/L 4HC and/or to 3 µmol/L VERT. Data are mean ± SD (*Bax*) and median ± interquartile range (IQR) (*Bcl2*). Relative expression of (**c**) *Bax* and (**d**) *Bcl2* after 48 hours of culture among the groups. Data are mean ± SD (*Bcl2*) and median ± interquartile range (IQR) (*Bax*). Ct were normalized on *Actin* and *Rpl19* levels, and on the mean Ct of the Control group. (N = 4). (*, $p < 0.05$). VERT, Verteporfin; 4HC, 4-hydroperoxycyclophosphamide.

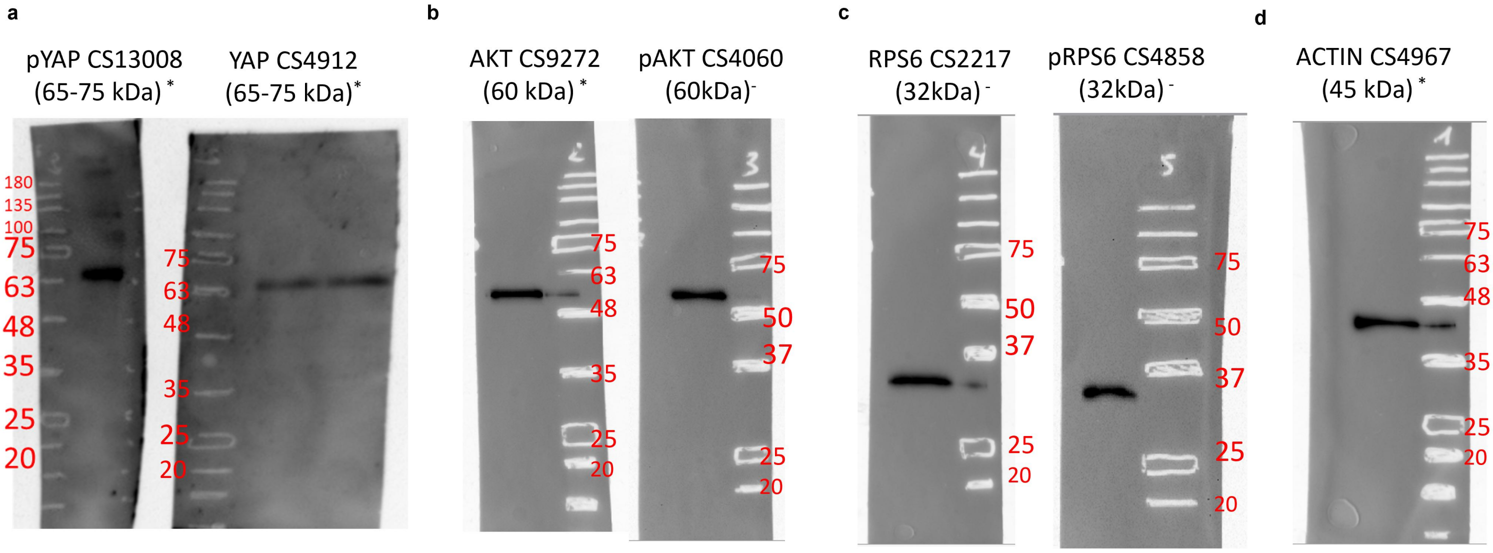

Supplementary Figure S7: Preliminary tests for antibodies validation on 8 PND3 mouse ovaries (fresh).

Western blot images of (**a**) pYAP and YAP, (**b**) AKT and pAKT, (**c**) RPS6 and pRPS6, and (**d**) ACTIN. 8 PND3 mouse ovaries in 200µL laemmli buffer were needed to achieve a signal, with 33µL protein extract volume spotted per well. Following validation of antibodies, blot membranes with cultured conditions were cut at expected molecular weight prior hybridization to allow the assessment of more than 6 proteins per sample. Ladder used: (*) Roti-Mark Tricolor Roth (#8271.1), and (-) Precision Plus Protein Dual Color Standards (#1610374) Biorad.

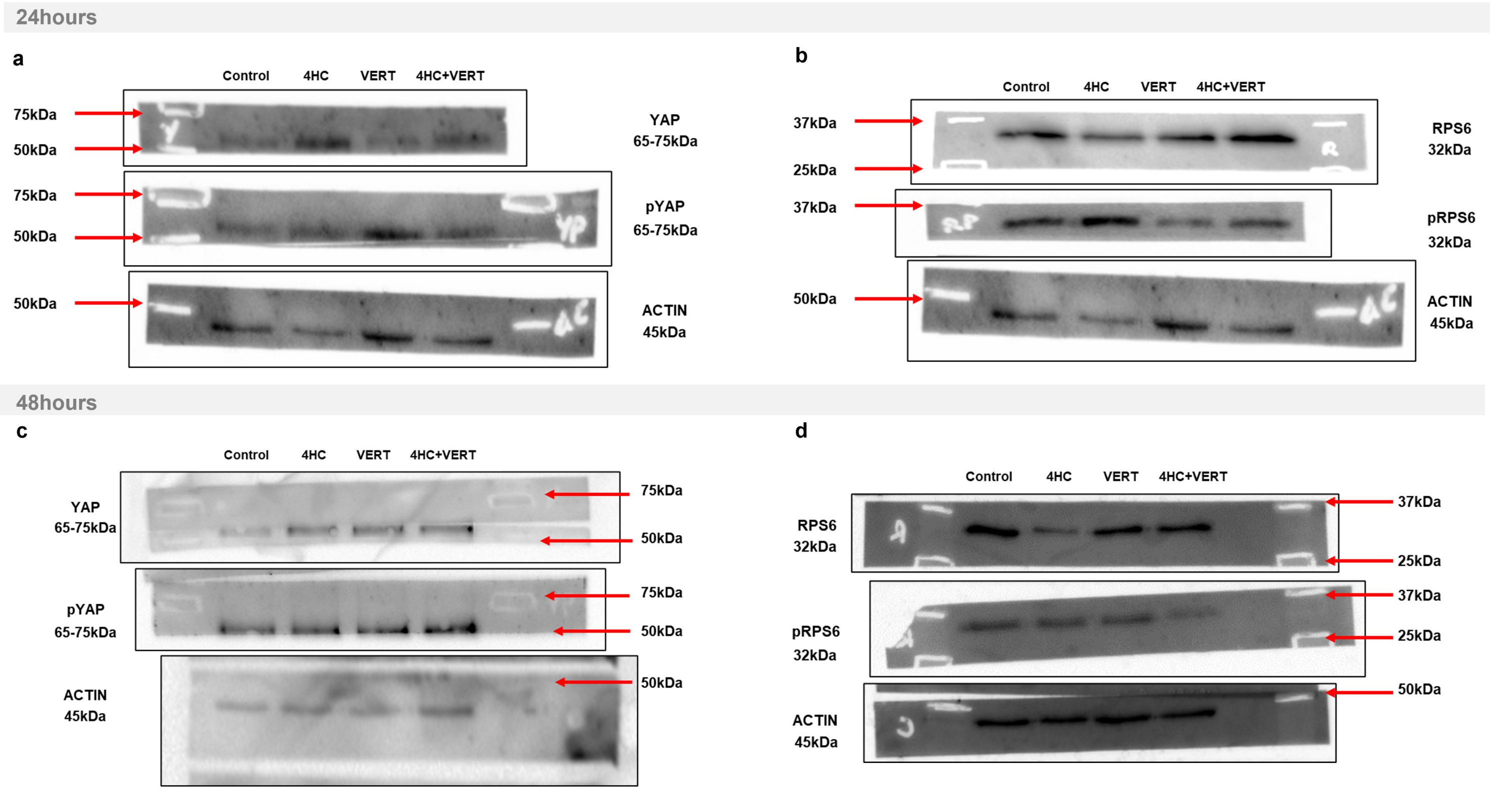

Supplementary Figure S8: Unprocessed blot images included in Figure 3 and 5 at higher exposure time.

Western blot images of YAP, pYAP and ACTIN levels at (**a**) 24 and (**c**) 48 hours of culture among the conditions. Protein blots of RPS6, pRPS6 and ACTIN at (**b**) 24 and (**d**) 48 hours of culture among the conditions. (N = 3). VERT, Verteporfin; 4HC, 4-hydroperoxycyclophosphamide.
